# Total laparoscopic transabdominal-transdiaphragmatic approach for treating Siewert II tumors: a prospective analysis of a case series

**DOI:** 10.1186/s12957-021-02136-2

**Published:** 2021-01-23

**Authors:** Wei Pang, Gang Liu, Yan Zhang, Yun Huang, Xinpu Yuan, Zhanwei Zhao, Chaojun Zhang

**Affiliations:** 1 Department of General Surgery, The Sixth Medical Center of PLA General Hospital, Beijing, 100048 China; 2 Department of General Surgery, Xinan Hospital, The Third Military Medical University, Chongqing, 400037 China

**Keywords:** Adenocarcinoma of the esophagogastric junction (AEG), Siewert II, Laparoscopic total gastrectomy

## Abstract

**Background:**

Although the morbidity of gastric cancer has decreased, the incidence of adenocarcinoma of the esophagogastric junction (AEG) is increasing. Furthermore, no consensus exists on which surgical approach should be applied for Siewert type II AEG. The purpose of our study was to evaluate the technical safety and feasibility of a new surgical approach.

**Methods:**

Sixty patients with Siewert type II AEG underwent laparoscopic total gastrectomy with the total laparoscopic transabdominal-transdiaphragmatic (TLTT) approach, which needs an incision in the diaphragm.

**Results:**

The median operative time, reconstruction time, and estimated blood loss were 214.8 ± 41.6 min, 29.40 ± 7.1 min, and 209.0 ± 110.3 ml, respectively. All of the patients had negative surgical margins.

**Conclusion:**

There were no intraoperative complications or conversions to open surgery. Our surgical procedure provides a unique option for the safe application of laparoscopic lower mediastinal lymph node dissection and gastrointestinal reconstruction.

**Trial registration:**

Chinese Clinical Trial Registry, ChiCTR1800014336. Registered on 31 December 2017 - Prospectively registered, http://www.chictr.org.cn/edit.aspx?pid=23111&htm=4.

**Supplementary Information:**

The online version contains supplementary material available at 10.1186/s12957-021-02136-2.

## Background

Adenocarcinoma of the esophagogastric junction (AEG) refers to adenocarcinoma that straddles the area of the esophagogastric junction (EGJ), including the distal esophagus and the proximal stomach, and is considered to have a high morbidity and low survival [1]. Furthermore, the incidence of AEG has increased rapidly in both Asian and Western countries [2]. Data from the Surveillance, Epidemiology, and End Results cancer registry program in the USA showed an approximate 2.5-fold increase in the incidence of AEG in the past 35 years. Moreover, the overall 5-year survival rates during this period were less than 12% [3]. As an East Asian region with a high incidence of GC, the statistical results of a Japanese cancer monitoring research group revealed that the proportion of AEG among all gastric adenocarcinomas increased from 2.3% in early 1960 to 10% in 2000, while the proportion of Siewert type II disease rose from 28.5 to 57.3%, but that of type I remained at approximately 1% [4]. In China, statistics from the single center of the West China Hospital of Sichuan University indicated that the proportion of AEG among all gastric adenocarcinomas has increased from 22.3 to 35.7% in the past 20 years [5].

Based on a retrospective review of 1602 consecutive resected patients [6], esophagectomy should be used for the treatment of type I tumors, while extended total gastrectomy should be adopted for the treatment of type II and type III tumors. In recent years, Western and Asian authors have reached a general agreement about the optimal surgical method for Siewert type I and III tumors, which is to apply total gastrectomy with distal esophagectomy with lower mediastinal lymphadenectomy and esophagectomy with a two-field Ivor Lewis operation via an exclusive abdominal approach.

However, no standard surgical method has been chosen for Siewert type II tumors to date. Subtotal esophagectomy with proximal gastrectomy through the transhiatal or transthoracic method or total gastrectomy with partial esophagectomy through a transhiatal approach is usually chosen. Takiguchi et al. [7] was the first in the world to report 6 cases of total laparoscopic transabdominal-transdiaphragmatic (TLTT) surgery and concluded that the diaphragmatic approach can provide a better surgical view for lymph node dissection and anastomotic reconstruction. However, due to the small sample size, the safety and effectiveness of this procedure still need to be further studied.

Thus, we conducted a single-center prospective study to further evaluate the safety and feasibility of the TLTT method for the treatment of Siewert II tumors. This study has been registered on the Chinese Clinical Trial Register (ChiCTR1800014336).

## Materials and methods

### Patients and specimens

This study was conducted at the Sixth Medical Center of PLA General Hospital. From February 2018 to January 2019, 60 consecutive patients suffering from AEG type II were chosen for this study. Endoscopy and computed tomography (CT) were included in the preoperative diagnostic evaluation. All the tumors covered in this study were histologically proven as AEG and defined as Siewert type II AEG. Tumor stage was classified based on the 8th edition of the TNM staging system of the International Union Against Cancer (UICC) for GC. Furthermore, the lymph node stations were numbered based on the definitions of the Japanese Gastric Cancer Association [8].

More than 200 laparoscopic gastrectomy procedures have been performed by the same surgical group each year. This group obtained professional training for laparoscopic surgery beforehand. The possible risks and complications were known to the patients. All patients provided written informed consent before the operation, and this study was approved by the ethics committee of our hospital (number of ethics approval: 2017011). The clinicopathological features of the patients are summarized in Table 1.

**Table 1:** Characteristics of the patients in this study (*N* = 60)

Sex	
Male	51 (85%)
Female	9 (15%)
Age (year)	66.10 ± 11.14
BMI (kg/m^2^)	22.98 ± 2.69
Tumor size (cm)	3.2 ± 0.9
Tumor classification	
Siewert type II AEG	60 (100%)
Siewert type III AEG	0 (0%)
Neoadjuvant chemotherapy	
−	9 (15%)
+	51 (85%)

*BMI* body mass index, *AEG* adenocarcinoma of the esophagogastric junction

### Surgical procedure

Typically, under general anesthesia, the patients were placed in a reverse Trendelenburg position with their legs apart. The umbilicus was chosen for the camera port, which can permit a flexible laparoscope to be introduced with a 10-mm trocar. Altogether, four other trocars were inserted into the upper abdomen. During this process, the assistant stood on the right side of the patient, while the surgeon usually stood on the left side of the patient. The video laparoscope operator filmed from a distal position and stood between the patients’ legs.

The abdominal cavity was explored and revealed no metastases in the greater omentum, peritoneum, free fluid, or liver. During the process of mobilizing the stomach, the left gastroepiploic artery (No. 4sb), the short gastric artery (No. 4sa), and other lymph nodes were removed. Subsequently, the suprapancreatic lymph nodes were excised. This led to the removal of the lymph nodes around the left gastric artery (No. 7), common hepatic artery (No. 8a, No. 8p), celiac artery (No. 9), and proximal splenic artery (No. 11p). The lower perigastric lymph nodes (No. 5, No. 6) were removed during routine D2 lymphadenectomy.

**Fig. 1:**
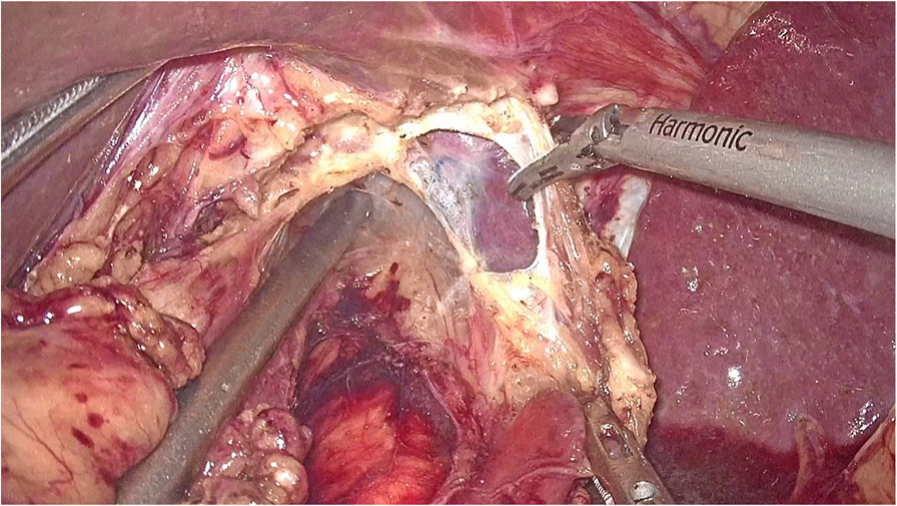
Left diaphragm was incised (5–7 cm)

**Fig. 2:**
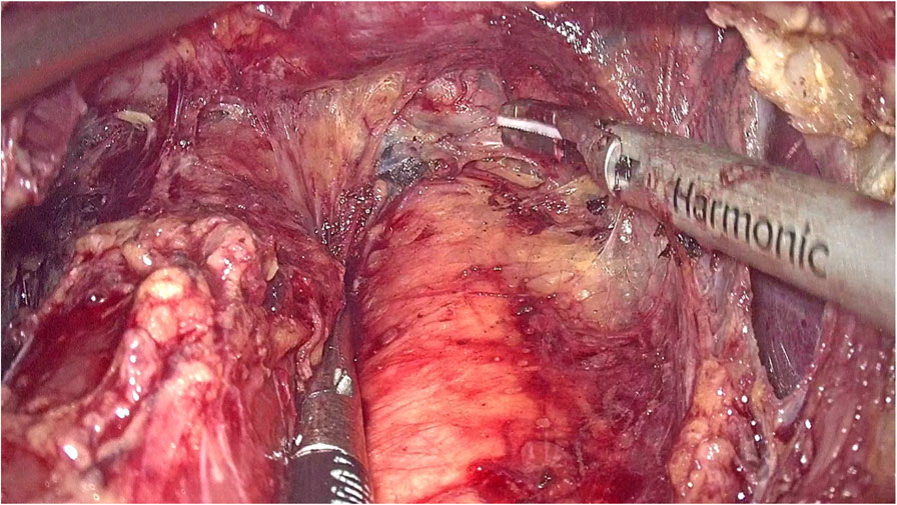
Lymph node dissection in the lower mediastinum

**Fig. 3:**
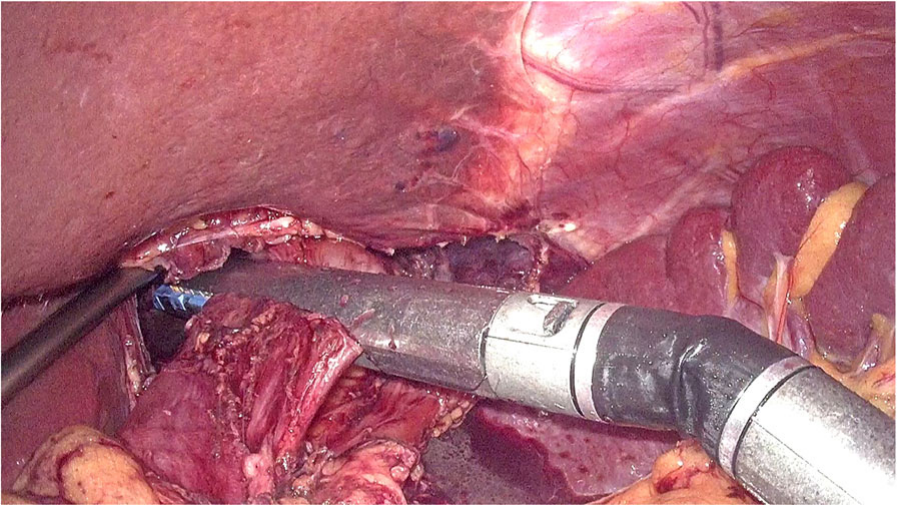
Esophagus was cut off through a 60-mm endoscopic linear stapler


The left gastric artery was classified and ligatured. Afterwards, the abdominal esophagus was exposed circumferentially by the phrenoesophageal ligament. Both sides of the crus were cut open to release the cardia esophagus. During the operation, the right pericardial (No. 1), left pericardial (No. 2), and lesser curvature (No. 3) lymph nodes were thoroughly removed. The laparoscopic dissection of the mediastinal lymph nodes was obstructed by the diaphragm around the lower esophagus. A 5–7-cm incision was made routinely in the diaphragmatic crus (Fig. 1, Supplementary video 1), and an abundant working space was created to enhance the view of the mediastinal space. After dissection of the muscle fibers of the esophageal hiatus cross-section, the thoracic aorta was exposed. The esophageal artery was classified and confirmed during dissection of the posterior layer of the esophagus. The dissection of the left side of the distal esophagus and anterior esophagus was conducted down to the level of the tracheal bifurcation. The surgeon incised the left parietal pleura close to the pericardium. Afterwards, the surgeon opened the left thoracic cavity to the mediastinal space. The incision was extended to the left pulmonary aortic arch and hilum, leading to a large surgical field. In this manner, the lower thoracic esophageal (No. 110), supradiaphragmatic (No. 111), and posterior mediastinal (No. 112) nodes were dissected (Fig. 2, Supplementary video 1). The transection plane of the esophagus was determined through intraoperative endoscopy. A 60-mm articulating endoscopic linear stapler was used to transect the esophagus 5 cm above the proximal margin (Fig. 3, Supplementary video 1). Intraoperative frozen pathology studies were routinely conducted.

**Additional file 1:.** Supplementary video 1.

#### Anastomosis method

The jejunum was cut off by 30 cm at the distal end of the ligament of Treitz; the stapler was inserted from the distal end. At the stump of the esophagus, a 25-mm circular stapler anvil head was placed, with hand-sewn purse-string sutures or a suture made with a needle through an esophagotomy. A circular stapler was used to perform the intracorporeal end-to-side esophagojejunostomy. The anastomosis could be made in the functional π-shaped anastomosis manner or overlap method (Supplementary video 2), in which one prongs of a linear stapler were inserted into the anterior wall of the jejunum via a small hole made 5–10 cm from the edge. Other prongs of a linear stapler were inserted into the posterior wall of the esophageal stump, and another hole was made. Meanwhile, side-to-side anastomosis was performed. Subsequently, a hand-sewn technique was used to close the common entry hole with a continuous suture. Repairing the hiatus of the diaphragm through a continuous suture is important to avoid hiatal hernia. The jejunum at 45–60 cm distal to the esophagojejunostomy was anastomosed with the input loop jejunum. A jejunal nutrition tube was inserted 45 cm distal to the second anastomosis. Two abdominal drainage tubes were placed in either side of the esophageal anastomosis.


**Additional file 2:.** Supplementary video 2.

### Statistical analysis

Statistical analyses were performed by using SPSS 21.0. Categorical data were compared by Fisher’s exact test or *χ*^2^ test. Consecutive data are presented as the mean ± standard deviation (SD). *T* tests or rank-sum tests were used to compare the means of two groups. *P* < 0.05 was considered statistically significant.

## Results

To date, these 60 patients have already successfully undergone TLTT resection for AEG type II. There were no patients of AEG type III. The tumor size was 3.2 ± 0.9 cm. Nine patients did not undergo preoperative chemotherapy. Table 1 presents the clinical features of the participants in this study.

All of these patients had negative surgical margins. The circular stapler was used in 27 patients, of whom 24 patients received hand-sewn purse-string sutures, and 3 patients received a suture with a needle through an esophagotomy. Additionally, functional end-to-end anastomosis was performed with a linear stapler in 24 patients. The overlap method was used in 9 patients. No conversions to open surgery were needed. The median operative time and reconstruction time were 214.8 ± 41.6 min and 29.40 ± 7.1 min, respectively. There were no reductions in oxygen saturation while the thoracic cavity was open in any patients with a pneumoperitoneum. Table 2 demonstrates the pathological and surgical findings.

**Table 2:** Surgical and pathological results (*N* = 60)

Type of surgery	
Total gastrectomy	60 (100%)
Operative time (min)	214.8 ± 41.6
Reconstruction time (min)	29.40 ± 7.1
Reconstruction type	
Using circular staplers	
Hand-sewn purse-string sutures	24 (40%)
Others	3 (5%)
Using linear staplers	
Functional end-to-end anastomosis	9 (15%)
Overlap method	24 (40%)
Length of esophageal exposure (cm)	9.4 ± 1.8
Number of lymph nodes dissected	37.3 ± 11.1
Blood loss (ml)	209.0 ± 110.3
Tumor depth (pathological)	
T1	9 (15%)
T2	15 (25%)
T3	15 (25%)
T4	21 (35%)
Nodal status (pathological)	
N0	24 (40%)
N1	9 (15%)
N2	6 (10%)
N3	21 (35%)
Histological type	
Differentiated	48 (80%)
Undifferentiated	12 (20%)

*TNM* tumor-nodes-metastasis

Ten patients had postoperative complications, including anastomotic fistula, anastomotic twist, pulmonary infection, incisional infection, and colon hernia (Table 3). One patient died as anastomotic fistula at postoperative 28 days. The follow-up approach was specifically designed for patients, which were conducted by telephone and at outpatient clinics. With one and a half years follow-up, one patient died due to pneumonia after developing multiorgan failure. One patient died as a consequence of anastomotic fistula. One patient died due to multiorgan failure following multiple metastases.

**Table 3:** Postoperative complications

Postoperative complications	10
Anastomotic fistula	3
Anastomotic twist	1
Pulmonary infection	4
Incisional infection	1
Hernia	1

## Discussion

The procedure of gastrectomy is usually chosen according to the size and location of tumors [9]. However, the approach has not been standardized so far. Theoretically, a safer upper surgical margin and better lower mediastinal lymph node dissection can be obtained through the transthoracic method. The elevated risk of perioperative pulmonary complications induced by thoracotomy can be prevented with the abdominal method. Hulscher et al. [10] implemented a randomized controlled trial to compare the abdominal/transhiatal approach and the IL approach for the treatment of adenocarcinoma of the gastric cardia and distal esophagus. There were no notable differences between these two groups in terms of the quality-adjusted survival, disease-free survival, or median overall survival. Wei et al. [11] performed a meta-analysis to compare transhiatal surgery and transthoracic surgery for AEG. No difference was shown in the general survival of patients with Siewert type II AEG between the two groups. However, transhiatal surgery would result in reduced pulmonary complications and a shortened hospital stay. As shown by the Japan Clinical Oncology Group (JCOG9502), the transhiatal method was superior to left thoracotomy in the treatment of AEG type II and III tumors with esophageal invasion depths of less than 3 cm [9].

The possibility of an improper proximal margin over the esophagus is one challenge in achieving R0 resection with tumors. A negative proximal margin of a minimum of 5 cm was advocated for by classic teaching. Barbour et al. [12] proposed that at least 3.8 cm isolated proximal surgical margins and 5 cm margins in situ should be included in the radical resection for T2+ stage AEG. Later, Mine et al. [13] demonstrated that a 2-cm proximal surgical margin in situ was sufficient for AEG. To date, no standard proximal surgical margin exists for Siewert type II AEG. Hosoda et al. [14] suggested that the lymph nodes of stations 1, 2, 3, and 7 can be dissected in Siewert type II and III AEG due to their high indexes of estimated benefit from lymph node dissection (IEBLDs). According to the report from Kurokawa et al. [15], metastasis is associated with Siewert type II AEG ≥ 2 cm. Patients suffering from esophageal invasion deeper than 3 cm should undergo dissection of the inferior mediastinal lymph nodes. Lee et al. [16] and Peng et al. [17] proposed that removing the mediastinal lymph nodes would help decrease the symptoms of advanced cancers. To date, no standard adequate lymphadenectomy approach exists.

In our study, the left diaphragm was opened to create a relatively large surgical field. High anastomosis in patients with esophageal invasion remains a technical challenge. The insertion of an anvil head in the esophagus is a major obstacle. A circular stapler was appropriate for the upper edge of tumors involving the distal esophagus (> 2 cm above the EGJ). The π-shaped anastomosis can be successfully used for the upper edge of tumors involving the distal esophagus (< 1 cm above the EGJ). The overlap approach was more appropriate for the upper edge of tumors involving the distal esophagus (1–2 cm above the EGJ). Thus, the overlap approach was the main anastomosis method that we chose for most of Siewert type II AEG [18–20].

In our study, the left diaphragm was generally split to obtain better exposure for reconstruction and lymph node dissection. When the reconstruction was conducted over the deeper mediastinum, a laparoscopic opening in the left diaphragm was helpful. A negative proximal margin of more than 5 cm was achieved in each patient. Thorough lymph node dissection was performed, including the thoracic esophageal nodes (No. 110), supradiaphragmatic nodes (No. 111), and posterior mediastinal nodes (No. 112). This preliminary study suggested that this operation mainly has the following advantages in comparison with the combined abdominal approach and traditional left thoracic approach (IL). First, surgeons and the patient could stay in fixed positions without changing the patient’s position during the operation, which obviously shortened the operation time and avoided turning the patient from a supine position. Second, the procedure was rather simple. Only a 10-cm incision was made laparoscopically on the diaphragm. Therefore, the surgical field could be expanded, which would be conducive to reducing the difficulty of anastomosis reconstruction and lymphatic dissection. Third, the dissection of suspicious lymph nodes and thorough resection of the surgical margin were achieved through this TLTT procedure. In the comparison with the traditional hiatal/transabdominal method or a transthoracic method, this procedure has the following advantages: shorter operation time, simple anastomosis operation, larger operation space, and better vision [21], with less pulmonary complications and less anastomotic leakage [22].

Although this surgical procedure was safe and feasible and the advances of this method were obvious, the limitations in our study should be recognized, including the single-center study and retrospective design, the lower number of clinical patients. Prospective clinical trials should be used to evaluate the benefits of this new surgical technique.

## Conclusions

Our prospective study showed that the TLTT method for AEG type II can be applied safely and is technically feasible after surgeons obtain proper experience in performing laparoscopic proximal gastrectomy and laparoscopic total gastrectomy. Long-term safety will be determined in follow-up studies. Nevertheless, this still remains a very advanced and complex laparoscopic procedure. The esophagojejunal anastomosis in the lower mediastinum is particularly demanding in terms of techniques. Future research should address the problems, such as type of anastomosis and standardization of operative techniques. It is recommended that this operation should be performed by experienced laparoscopic surgeons.

## Data Availability

The datasets used and analyzed during the current study are available from the corresponding author on reasonable request.

## Abbreviations

AEG: Adenocarcinoma of the esophagogastric junction
GC: Gastric cancer
EGJ: Esophagogastric junction
TLTT: Total laparoscopic transabdominal-transdiaphragmatic
